# Evaluation and assessment of prescribing patterns in elderly patients using two explicit criteria based screening tools: (The PRISCUS list and STOPP/START criteria)

**DOI:** 10.12669/pjms.346.14928

**Published:** 2018

**Authors:** Irum Butool, Shabnam Nazir, Maryam Afridi, Syed Majid Shah

**Affiliations:** 1 *Irum Butool, Department of Pharmacy, Kohat University of science and Technology, Kohat, Pakistan*; 2 *Shabnam Nazir, Assistant Professor, Department of Pharmacy, Kohat University of science and Technology, Kohat, Pakistan*; 3 *Maryam Afridi, Department of Pharmacy, Kohat University of science and Technology, Kohat, Pakistan*; 4 *Syed Majid Shah, Department of Pharmacy, Kohat University of science and Technology, Kohat, Pakistan*

**Keywords:** Potentially inappropriate medication, Potential prescribing omissions, PRISCUS list, STOPP/START criteria, Ambulatory elderly

## Abstract

**Background & Objectives:**

Rational prescribing can prevent medication errors and the associated harm, especially in old age patients, as they are being frequently prescribed with drugs for various ailments. Moreover, polypharmacy is a common practice in them. Therefore, a significant threat of potential drug interactions and adverse effects exist. Current study focuses on assessment of Potentially Inappropriate Medication (PIM) in medication prescribed to old age patients.

**Methods:**

It was a forty days, descriptive and observational study conducted from August 15^th^ 2017 to September 25^th^ 2017 in which prescriptions given to elderly patients were reimbursed for collecting various sets of information. In order to assess PIMs (in Pakistani Set-up), STOPP/START addition 2008 (including examples of misprescribing, overprescribing and under prescribing) and the PRISCUS list (misprescribing and overprescribing) was used. Statistical analysis of results was performed using SPSS version 20.

**Results:**

One hundred forty six cases of PIMs (including incorrect prescribing, overprescribing and under prescribing) were detected. It included incorrect prescriptions 104, under prescription 28 and over prescriptions 14. NSAIDS accounted for most incorrect prescriptions followed by benzodiazepines. Mostly NSAIDS were used for myalgia, backache and rheumatoid disorders.

**Conclusion:**

Current findings highlighted Potentially Inappropriate Prescribing (PIP), particularly of NSAIDs and under prescribing of statins in cardiovascular diseases. Study findings suggest introducing pertinent interventions at the stages involved in prescribing, prescription review and its follow up to reduce the PIP and PIMs.

## INTRODUCTION

Prescribing quality is an important cognitive factor for determining the wellbeing of elderly population while increased demand of an effective prescribing is a challenge for primary health care practitioners.^1^ Polypharmacy in elderly is a common clinical practice because they have several concomitant diseases.

Inappropriate medication prescribing can lead to an increased risk of Adverse Drug Events (ADE) ranging from minor to serious life threatening i.e. Myocardial Infarction (MI), ventricular reentry arrhythmias etc.^2^,^3^ Medications like CNS depressants, antipsychotics, Tricyclic Antidepressants (TCA), selective serotonin reuptake inhibitors (SSRIs),^4^ Non-Steroidal Anti-Inflammatory Drugs (NSAIDs) and anticholinergic agents are associated with increased risk of falling (50%), hip fracture, gastrointestinal bleeding, cognitive impairment and functional decline.^5^ Therefore Potentially Inappropriate Prescribing (PIP) is highly prevalent particularly in elderly and is an important concern for patient safety and rational health care services.^6^

A Potentially Inappropriate Medication (PIM) list is a useful tool for elderly in preventing adverse drug events occurring at prescription stage. Potentially Inappropriate Medication (PIM) highlights Potentially Inappropriate Prescribing (PIP), Potential Prescribing Omissions (PPO),^7^ potentially harmful drug-drug/drug-disease interactions i.e. using NSAIDs in patients with hypertension,β blocking agents in diabetic patients,^5^,^8^ Poly pharmacy, morbidity, contraindications based on patients genetic profile and those with age related contraindications.^9^

In recent years, several PIM lists have been published using various screening tools.^10^,^11^ The most widely used Beers criteria, was developed by expert panel in 1991.^9^ Despite the fact that it is frequently cited and used world widely,^11^ some of its aspects have been criticized with regard to drug scope and its adaptability in relation to European prescribing pattern.

A new PIM list termed as PRISCUS list was defined by Holt et al specifically to be used for evaluating geriatric pharmacotherapy in Germany.^12^ Out of 18 drug classes, a total of 83 drugs were rated as Potentially Inappropriate Medications (PIMs) in the list.^12^,^13^

Furthermore; a systems defined as medication review tool termed as STOPP (Screening Tool of Older Person’s Prescription) and START (Screening Tool to Alert doctors to Right Treatment) criteria introduced in 2003, detects the dual nature of inappropriate prescribing i.e. PIMs (using STOPP) and Potential Prescribing Omissions (PPOs) (using START).^14^

Current study aimed to detect PIMs in geriatric pharmacotherapy using three sets of published explicit criteria STOPP/START criteria and PRISCUS tool. This study also explores the prevalence rates of Potential Inappropriate Prescribing (PIP) and Potential Prescribing Omissions (PPO) at ambulatory health care setting.

## METHODS

Current study was descriptive and observational. It covered reimbursed medications prescribed to a set of ambulatory elderly patients (age range = 50-87 years old) who accessed outpatient pharmacy, of a tertiary care hospital, KPK, Pakistan were analyzed. The study was conducted over a period of forty days (15 August 2017- 25 September 2017). It was made sure that the selected pharmacy serves customers of wide range in terms of age and diagnosis. Median number of medication in each was n = 3. During data collection nonprescription medications and natural products weren’t considered.

Variable levels of information related to diagnosis and patients demographics were collected through prescriptions. PIMs list as a result of collected information was the reflective of medicines being used in Pakistan. The analysis used three sets of PIM identification criteria: STOPP/START, addition 2008 (including examples of misprescribing = erroneous mode of prescribing that violates standard way of prescribing, overprescribing = prescribing a drug in greater amounts or on more occasions than the appropriate prescribing and under prescribing = prescribing pattern less than appropriate) and the PRISCUS list (incorrect prescribing and overprescribing). Both sets of criteria’s are applicable in certain situations based upon the availability of medical history and the information of laboratory biochemical tests. PRISCUS list is much better to apply in situations when the usually needed clinical information. Both of the evaluation criteria’s cover most of the medications and classes of drugs and were adequate for descriptive nature of study. Patient’s privacy was fully observed during analysis of data however, the prior consent wasn’t obtained as the study was retrospective and observational in nature. Study was conducted under approved clinical clerkship program by department of Pharmacy, Kohat University of Science and Technology. A data collection form was designed to collect the data (Index is attached). Statistical analysis of results was performed using SPSS version 20.

## RESULTS

Among 300 prescriptions 138 (46%) to men and 162 (54%) were prescribed to women. Highest number of elderly patients suffered from rheumatologic disorder (90 (20.41 %) followed by urologic (56 (12.7%)), cardiovascular 46 (10.43%), respiratory 22 (5%) and then other complications (Table-I).

**Table-I:** Patients’ characteristics.

	Data Collection: Prescriptions of reimbursed medications:
Items of data included in each collection:	300 prescriptions
Mean age±SD (years old)	60.16 (±8.165)
Range of individual age	50-87
Men (% of the elderly patients in each population groups)	138 (46%)
Women (% of the elderly patientsin each population groups)	162 (54%)
Age groups:	
46-60 years old	189
61-75 years old	95
>76years old	16
No of medications in each collection of data±SD	1262 (1.353%)
Total number of diagnosis	441
Frequency of types of diagnosis in each collection of data:	
*Type of diagnosis*	*No. of prescriptions with the respective type of diagnosis*
Cardiovascular (CVS)	46 (10.43%)
Neuropsychiatric	13 (2.95%)
Rheumatologic	90 (20.41 %)
Diabetes	21 (4.76%)
Gastrointestinal (GIT)	18 (4.081%)
Respiratory	22 (5%)
Urologic	56 (12.7%)
Ophthalmologic	04 (0.907%)
Other	41 (9.3%)

Total Number of PIMs found was 146. Incorrect prescribed PIM accounted for 104 (71.23%) which was found to be more frequent followed by under prescribed 28 (19.18%) and then overprescribed PIM 14 (9.59%) (Table-II).

**Table-II:** Types of Potentially Inappropriate Medications (PIM).

Medications sample analyzed:	
–Total number of medications prescribed to the ambulatory elderly:	1262
–Total number of PIMs in each sample of medications prescribed:	146
Subtype – PIM:	
Misprescribed – PIM:	104 (71.23%)
Underprescribed– PIM:	28 (19.18%)
Overprescribed– PIM:	14 (9.59%)

**Table-III:** Potentially inappropriate medications identified in the ambulatory elderlypharmacotherapy.

Subtype – PIM Reference	no. of examples	% of the respective	% of 146
Criteria: reason to avoid	of subtype-PIM	subtype-PIM	PIM
Misprescribed – PIM	104		
NSAIDs as chronic analgesics in myalgia, backache and rheumatoid diseases:	49	47.11%	33.56%
PRISCUS list –analgesics, anti-inflammatory drugs:			
– very high risk of gastrointestinal hemorrhage, ulceration, or perforation, which may be fatal			
– indometacin: central nervous disturbances			
– phenylbutazone: blood dyscrasia			
– etoricoxib: cardiovascular contraindications			
STOPP tool –musculoskeletal system	15	14.42%	10.27%
– NSAID with moderate-severe hypertension	14		
– Long-term NSAID or colchicine for chronic treatment of gout where there is no contraindication to allopurinol	01		
Benzodiazepines:	12	11.54%	8.22%
PRISCUS list - sedatives, hypnotic agents:			
Long, short and intermediate acting:			
– risk of falling with risk of hip fracture			
– prolonged reaction times			
– psychiatric reactions			
– cognitive impairment			
PRISCUS list - (diphenhydramine)	03	2.88%	2.05%
– anticholinergic effects			
– dizziness			
– ECG changes			
Anticholinergic drugs (Antihistamines)	02	1.92%	1.37%
PRISCUS list- Anticholinergic drugs:			
– anticholinergic side effects (e.g., constipation, dry mouth)			
– impaired cognitive performance			
– ECG changes (prolonged QT)			
STOPP tool –CNS and Psychotic Drugs	03	2.88%	2.05%
fluazepam, nitrazepam, chlorazepate and benzodia- zepines with long-acting metabolites e.g. diazepam	02		
–Prolonged use (> 1 week) of first generation antihistamines i.e. diphenhydramine, cyclizine, chlorpheniramine, promethazine	01		
Antipsychotics:	01	0.96%	0.68%
PRISCUS- neuroleptic drugs:			
– anticholinergic and extrapyramidal side effects			
– clozapine: increased risk of agranulocytosis and myocarditis			
STOPP tool – CNS and Psychotropic Drugs	01	0.96%	0.68%
–neuroleptics as long-term hypnotics	01		
Antiemetic drugs:	02	1.92%	1.37%
PRISCUS list - Tricyclic antidepressants:			
– peripheral and central anticholinergic side effects			
– cognitive deficit – increased risk of falling			
STOPP tool – CNS and Psychotropic Drugs	02	1.92%	1.37%
PRISCUS list – (dimenhydramine)			
– anticholinergic side effects			
Anti-dementia drugs, vasodilators, circulation-promoting agents:	01	0.96%	0.68%
PRISCUS list – (piracetam)			
– no proof of efficacy, unfavorable risk/ benefit profile			
Duplicate drug classes (two concurrent NSAIDs)	10	9.61%	6.85%
Endocrine System:	01	0.96%	0.68%
Underprescribed – PIM	28		
Underprescribing statins in coronary, cardiovascular and cerebrovascular diseases:	13	46.43%	8.90%
START tool - Cardiovascular system:			
–Statin therapy			
Underprescribing of statins in diabetes mellitus:	01	3.57%	0.68%
START tool - Endocrine system:			
–Statin therapy in diabetes mellitus if additional cardiovascular risk factors present			
Underprescribing of antihypertensive therapy where systolic blood pressure consistently>160 mmHg	03	10.71%	2.05%
Underprescribing of B-2 agonists or anticholinergic agents in asthma:	01	3.57%	0.68%
Underprescribing antidepressants in the presence of mild-moderate depressive symptoms lasting at least 3months:	02	7.14%	1.37%
UnderprescribingCa and Vitamin D supplements in patients with known osteoporosis:	03	10.71%	2.05%
Underprescribing DMARD with moderate-severe rheumatoid disease lasting > 12 weeks	02	7.14%	1.37%
Underprescribing metformin with type 2 diabetes:	01	3.57%	0.68%
Underprescribing ACE inhibitor or Angiotensin Receptor Blocker in diabetes with nephropathy:	01	3.57%	0.68%
Underprescribing L-DOPA in idiopathic Parkinson’s disease:	01	3.57%	0.68%
Overprescribed – PIM:	14	100%	9.59%
Overprescribing NSAIDs			
– very high risk of gastrointestinal hemorrhage, ulceration, or perforation, which may be fatal	14		

Among the various classes of drugs, NSAIDs ratio dominated in the category of incorrect prescribing PIM 47.11 %, followed by benzodiazepines and others. Their main diagnosis for which NSAIDs were used included myalgia,^15^ backache and rheumatoid diseases. Among the various NSAIDs, frequency of use of acetaminophen was highest followed by diclofenac sodium and then others in collected data of elderly patients. NSAIDs were co prescribed majorly with cardiovascular medication. Their use was chronic, as they were being used for more than a month while proton pump inhibitors were also not used concurrently.

Benzodiazepines use was at second place in the category of incorrect prescriptions PIM with 11.54%. In elderly patients use of short to long-acting benzodiazepines for daily use was a common phenomenon. Psychotropic medication was 2.88 % in the category of incorrect prescriptions PIM and it was also for a prolonged use > 1 week.

The statins was the leading class of drugs in the incorrect prescriptions PIMS category 46.43%, followed by antihypertensive drugs specifically beta 2 agonists or anticholinergic in asthmatic conditions. Overprescribed PIMs category was mostly represented with NSAIDS.

## DISCUSSION

Current study has successfully evaluated various types of PIMs in a prescription data set of elderly patients by utilizing screening tools like the STOPP/START criteria and PRISCUS list. In total 300 prescriptions, the number of medications prescribed for various diagnosis were 1262 and were exposed to a total 146 PIMs categorized as misprescribed (71.23%), underprescribed (19.18%) and overprescribed (9.59%) PIMs. Use of PRISCUS and STOPP/START criteria are carefully selected assessment criteria which made study results more authentic.^5^ At most of the time the clinical details of elderly patients were unavailable so PRISCUS tool proved effective at those places.

This is a pilot study which assesses PIMs specifically in elderly and is also the first study conducted at a local community level in Pakistan by using two explicit criteria combinedly i.e. PRISCUS and STOPP/START criteria. It is suggested to extend current study on a larger scale and covering a prolonged period. This study also highlights the importance of geriatric dose optimization and healthcare to be very important part of pharmaceutical care.

Nearly, 71.23% of the incorrect prescriptions PIMs detected by STOPP and PRISCUS tool in our study involved six categories of medicines i.e. NSAIDs, Benzodiazepines, Anticholinergic, Anti-dementia drugs, CNS and Psychotropic Drugs. According to another study, in institutionalized elderly frequent use of anticholinergic, long-acting benzodiazepines and psychotropics was found.^16^ Potential inappropriate prescribing (PIP) of sedatives, hypnotics and anticholinergics is associated with, in particular increased risk of falling, hip fracture, psychiatric reactions, ECG changes (prolonged QT) and cognitive impairment.^12^ Thus it is suggested that long term sedative and hypnotic agents should be avoided in elderly. Our study also detected duplicate drug class prescriptions (7%) using STOPP tool which can intensify side effects, thereby highlighting the need to develop pharmaceutical screening programme to support rational drug prescribing.

In this study, the number of overprescribed-PIMs was associated with NSAIDs use (10%). Several studies report hazards associated with multiple NSAIDs therapy i.e. Gastrointestinal (GI) complications and cardiovascular risks.^17^ Current study showed that multiple NSAIDs prescription in elderly was a common practice in most of the cases. However few prescribed NSAIDs were without any clear indication. NSAIDs induced Gastrointestinal (GI) complications can be prevented by co-administering PPIs.^18^ Despite the known association of NSAIDs induced gastropathy, it was found that PPIs were co-administered to only about 18.33 % of the patients. Furthermore the number of Potential Prescribing Omissions (PPOs) found in our study were 28 (19.18%). One of the study highlighted over prescription of statins,^19^ however, in our study, START tool demonstrated that statins use was associated with under prescribed-PIMs (9%). A review study including observational data of retrospective studies based on STOPP/START mentioned indeterminate frequency of PIMs ranging from 21.4% to 79% and few results were quite comparable to the current study.^6^ Therefore both STOPP/START and PRISCUS may be considered more appropriate detection tools for identifying prescribing errors in elderly which leads to Adverse Drug Event (ADE) and are associated with iatrogenic morbidity.

## CONCLUSION

This study has highlighted potentially inappropriate prescribing (PIP) of NSAIDs as chronic analgesics in myalgia, backache and rheumatoid diseases, duplicate drug class prescriptions and under prescribing of statins in cardiovascular diseases. These findings stimulate a need to develop pharmaceutical screening programme to rationalize prescribing practices in geriatric pharmacotherapy and for conducting further research in the same area covering large population.

### Author’s contribution

**IB:** Design, data collection and manuscript writing.

**SN:** Conceived, designed and was involved in data analysis and manuscript editing.

**MA:** Data collection.

**SMS:** Data analysis and manuscript editing.
